# A Proposed Modified Staging System for Medullary Thyroid Cancer: A SEER Analysis With Multicenter Validation

**DOI:** 10.1093/oncolo/oyad165

**Published:** 2023-06-13

**Authors:** Zhengshi Wang, Xin Fan, Xiaojuan Zha, Yong Xu, Zhiqiang Yin, Youlutuziayi Rixiati, Fei Yu

**Affiliations:** Thyroid Center, Shanghai Tenth People’s Hospital, Tongji University School of Medicine, Shanghai, People’s Republic of China; Shanghai Center of Thyroid Diseases, Shanghai Tenth People’s Hospital, Tongji University School of Medicine, Shanghai, People’s Republic of China; Department of Nuclear Medicine, Shanghai Tenth People’s Hospital, Tongji University School of Medicine, Shanghai, People’s Republic of China; Institute of Nuclear Medicine, Tongji University School of Medicine, Shanghai, People’s Republic of China; Shanghai Center of Thyroid Diseases, Shanghai Tenth People’s Hospital, Tongji University School of Medicine, Shanghai, People’s Republic of China; Department of Endocrinology and Metabolism, Shanghai Tenth People’s Hospital, Tongji University School of Medicine, Shanghai, People’s Republic of China; Department of Laboratory, Yueyang Hospital, Hunan Normal University, Yueyang, People’s Republic of China; Thyroid Center, Shanghai Tenth People’s Hospital, Tongji University School of Medicine, Shanghai, People’s Republic of China; Shanghai Center of Thyroid Diseases, Shanghai Tenth People’s Hospital, Tongji University School of Medicine, Shanghai, People’s Republic of China; Department of Pathology, Fudan University Huashan Hospital, Shanghai, People’s Republic of China; Department of Nuclear Medicine, Shanghai Tenth People’s Hospital, Tongji University School of Medicine, Shanghai, People’s Republic of China; Institute of Nuclear Medicine, Tongji University School of Medicine, Shanghai, People’s Republic of China

**Keywords:** medullary thyroid cancer, AJCC, staging, SEER, prognosis

## Abstract

**Background:**

The 8th edition of the American Joint Committee on Cancer (AJCC) staging system for medullary thyroid cancer (MTC) was implemented in 2018. However, its ability to predict prognosis remains controversial.

**Patients and Methods:**

Patient data were obtained from the Surveillance, Epidemiology, and End Results (SEER) database and multicenter datasets. Overall survival was the primary end-point of the present study. The concordance index (C-index) was used to assess the efficacy of various models to predict prognostic outcomes.

**Results:**

A total of 1450 MTC patients were selected from the SEER databases and 349 in the multicenter dataset. According to the AJCC staging system, there were no significant survival differences between T4a and T4b categories (*P* = .299). The T4 category was thus redefined as T4a’ category (≤3.5 cm) and T4b’ category (>3.5 cm) based on the tumor size, which was more powerful for distinguishing the prognosis (*P* = .003). Further analysis showed that the T category was significantly associated with both lymph node (LN) location and count (*P < .*001). Therefore, the N category was modified by combining the LN location and count. Finally, the above-mentioned novel T and N categories were adopted to modify the 8th AJCC classification using the recursive partitioning analysis principle, and the modified staging system outperformed the current edition (C-index, 0.811 vs. 0.792).

**Conclusions:**

The 8th AJCC staging system was improved based on the intrinsic relationship among the T category, LN location, and LN count, which would have a positive impact on the clinical decision-making process and appropriate surveillance.

## Teaser

This study investigated the intrinsic relationship and prognostic value of the T and N categories of the AJCC staging system using the SEER and multicenter databases. Results might provide a novel breakthrough for the improvement of the AJCC staging system for medullary thyroid cancer.

**Implications for Practice**

The 8th edition of AJCC staging system for medullary thyroid cancer was implemented in 2018; however, its ability to predict prognosis remains controversial. Here, it was found that the T category was significantly associated with both LN location and count. The AJCC staging system was therefore modified based on their intrinsic relationship, which was shown to have a stronger discriminatory capability.

## Introduction

Medullary thyroid cancer (MTC) belongs to neuroendocrine tumors and derives from para-follicular cells (C cells) of the thyroid gland. MTC is relatively infrequent, accounting for approximately 2% of all thyroid malignancies.^1^ Currently, surgery is the primary treatment for MTC.^2^ Despite significant improvements in comprehensive MTC treatment, such as immunotherapy and targeted therapies,^3-6^ the prognosis of MTC patients remains bleak, and MTC mortality can account for up to 13% of thyroid cancer-related deaths.^7^ There is therefore a significant need for accurate staging of MTC patients to improve the clinical assessments and patient management.

At present, the Union for International Cancer Control/American Joint Committee on Cancer (UICC/AJCC) tumor-node-metastasis (TNM) staging system is the most frequently used classification for MTC.^8^ However, its ability to predict prognosis remains controversial. Adam et al^9^ found that the current AJCC staging system (8th edition) did not significantly differentiate between patients with stages I, II, and III (*P* > .05) using data from The National Cancer Database (NCDB) and the Surveillance, Epidemiology, and End Results (SEER) database. Moreover, patients with stage IV had a much worse prognosis than those with stages I, II, and III. The 5-year survival rate of MTC patients with stage IV was merely 33% based on the SEER data while that of patients with stages I, II, and III was greater than 90%. Chen et al^10^ incorporated the metastatic lymph node ratio (LNR) into the staging system and the performance of the novel N staging system was improved to some extent. However, LNR was susceptible to many factors such as the number of resected LNs, pathological examination, and individual differences.^11,12^ There were also claims of integrating an innovative idea known as mortality per 1000-person-years into the staging system, which resulted in a great discrepancy in the stage distribution.^13^ 835 cases (87.25%) were classified into stages I-II while 122 (12.25%) were classified into stages III-IV. Additionally, the incorporation of mortality per 1000-person-years in the AJCC staging system was not a routine practice either. Although there had been some other studies trying to improve the AJCC staging system, the results were still unsatisfactory.^14,15^ Therefore, it is imperative to develop a more effective and rational clinical staging system.

In the previous study, we modified the staging system by including the LN count instead of the LN location.^16^ Here, we further investigated the prognostic values of the T category and N category and their intrinsic relationship using the SEER and multicenter databases. These might provide a novel breakthrough for the improvement of the staging system for MTC.

## Materials and Methods

### Data

The SEER database, one of the largest clinical databases in the US, was used to acquire patient data. The primary inclusion criteria were: (1) The age was over 18 years old. (2) The diagnosis of MTC was confirmed by histological evidence. (3) The only or first primary tumor was MTC. (4) TNM information is definite according to the 8th edition of AJCC TNM classification. The N1a category refers to metastasis to level VI or VII LN, and the N1b category refers to metastasis to the lateral or retropharyngeal LN. (5) The number of dissected LNs was greater than zero. (6) The number and location of dissected and positive LNs were definite. (7) Tumor size of patients with T4N*any*M0 was definite. (8) There was follow-up data available. M0 patients with not otherwise specified (NOS) information, including N1 and T4 NOS, were eliminated. If patients had distant metastasis, information regarding their T and N categories was not necessary because they were all classified into stage IV. The maximum follow-up period was 143 months.

The multicenter data was collected from Shanghai Tenth People’s Hospital, Xuzhou Central Hospital, Yueyang Hospital, and Changhai Hospital (2010-2018). All the participants were Chinese. The inclusion criteria and exclusion criteria were aligned with the SEER database. The maximum follow-up period was 94 months. Written informed consent was obtained from all participants. The present study followed STROBE reporting guidelines^17^ and was approved by the Bioethics Committee of Shanghai Tenth People’s Hospital, Tongji University School of Medicine, P. R. China.

### Statistical Analysis

The comparison of sample rates was performed by Chi-square test. Survival differences were estimated by the Kaplan-Meier method and reported as hazard ratio (HR) with 95% CI. The concordance index (C-index) was used to assess the efficacy of various models to predict prognostic outcomes. The above statistical analyses were performed using R software (Version 3.6.2). The optimal cutoff values of tumor size and LN count were determined by X-tile software, which was able to try every cutoff value and select the best one automatically. *P*-values less than .05 were considered statistically significant.

## Results

### Baseline Information

Based on the inclusion and exclusion criteria, a total of 1450 MTC patients were selected from the SEER databases between 2004 and 2017 (Table 1). The age of the entire cohort ranged from 18 to 99 years old and the median age was 53 years old. The proportion of female patients was slightly higher than that of male patients (58.7% vs. 41.3%). More than 80% of patients were White. Tumor size ranged from 1 to 150 mm with a median size of 24 mm. Regarding patient staging, T1 patients accounted for the most (38.8%) and T4 accounted for the least (9.8%). Among T4 patients, 95 cases were classified into the T4a category and 47 cases were classified into T4b. The number of positive LNs ranged from 0 to 73 with the median number of one. Among patients with LN metastasis (LNM), 224 cases were classified into the N1a category and 532 cases were classified into N1b. There were 395 cases with 1-8 positive LNs and 358 with >9 LNs. A total of 117 patients developed distant metastasis, accounting for 8.1% of all patients. Grade information was poorly documented and the overwhelming majority of patients (90.9%) were missing. However, it would not affect the following analysis and conclusions.

**Table 1.** Baseline information of entire cohort.

Variables	SEER (%)	Multicenter (%)
Total number	1450	349
Age (years)		
Median(range)	53 (18-99)	56(19-76)
Gender		
Male	599 (41.3%)	154 (44.1%)
Female	851 (58.7%)	195 (55.9%)
Race/ethnicity		
White	1204 (83.0%)	0
Black	131 (9.0%)	0
Others^*^	115 (8.0%)	349 (100%)
Grade		
I+II	78 (5.4%)	/
III+IV	53 (3.7%)	/
Unknown	1319 (90.9%)	/
TNM (8th)		
I	392 (27.1%)	65 (18.7%)
II	277 (19.1%)	46 (13.2%)
III	192 (13.2%)	87 (24.9%)
IV		
IVA	447 (30.8%)	95 (27.2%)
IVB	25 (1.7%)	19 (5.4%)
IVC	117 (8.1%)	37 (10.6%)
T category		
T1	563 (38.8%)	93 (26.7%)
T2	376 (26.0%)	72 (20.6%)
T3	359 (24.7%)	90 (25.8%)
T4a	95 (6.6%)	43 (12.3%)
T4b	47 (3.2%)	23 (6.6%)
TX	10 (0.7%)	28 (8.0%)
Tumor size (mm)^**^		
Median(range)	24 (1-150)	20 (3-85)
N category		
N0	679 (46.8%)	133 (38.1%)
N1a	224 (15.4%)	102 (29.2%)
N1b	532 (36.8%)	92 (26.4%)
N1 NOS	10 (0.7%)	0
NX	5 (0.3%)	22 (6.3%)
Number of positive LNs		
Median(range)	1 (0-73)	2 (0-42)
0	679 (46.8%)	133 (38.1%)
1-8	395 (27.3%)	104 (29.9%)
≥9	358 (24.7%)	86 (24.6%)
Unknown	18 (1.2%)	26 (7.4%)
M category		
M0	1333 (91.9%)	312 (89.4%)
M1	117 (8.1%)	37 (10.6%)

^*^Including American Indian/AK Native, Asian/Pacific Islander/Unkonwn in the SEER database and Chinese in multicenter dataset.

^**^Twenty-six cases not available in the SEER database.

Abbreviations: TNM, tumor-node-metastasis; LN, lymph node; SEER, Surveillance, Epidemiology, and End Results.

### Modification of T4 Category

**Figure 1.**
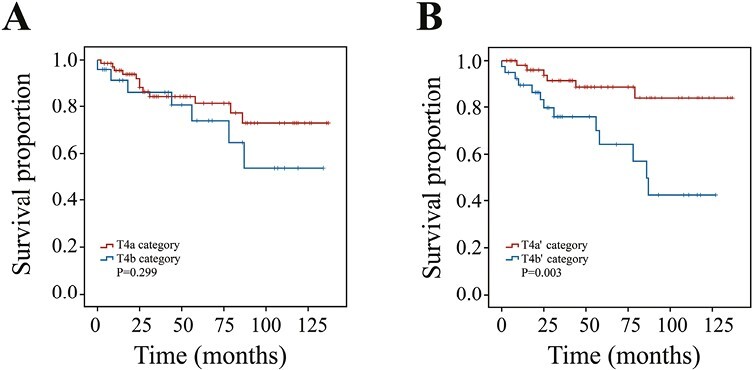
Kaplan-Meier survival curves stratified by the T4 category. (**A**) Survival curves of the T4a and T4b categories in the SEER database; and (**B**) survival curves of the T4aʹ and T4bʹ categories in the SEER database.

In the current staging system, the T4 category was divided into T4a (invading subcutaneous soft tissues, larynx, trachea, esophagus, or recurrent laryngeal nerve) and T4b (invading prevertebral fascia or encasing carotid artery or mediastinal vessels) categories according to the degree of tumor invasion. However, there were no significant survival differences between T4a and T4b categories (M1 patients excluded) (Fig. 1A, *P* = .299). The impact of tumor size was examined on the T4 category using X-tile software and we found that patients with a size >3.5 cm had a much worse prognosis than those with a size ≤3.5 cm (Fig. 1B, *P* = .003, HR = 3.941, 95%CI, 1.493-10.401). Thus, patients with size ≤3.5 cm were redefined as the T4a’ category, and patients with size >3.5 cm were redefined as the T4b’ category.

### Modification of N Category Based on the Intrinsic Relationship Among T Category, LN Location, and LN Count

**Figure 2.**
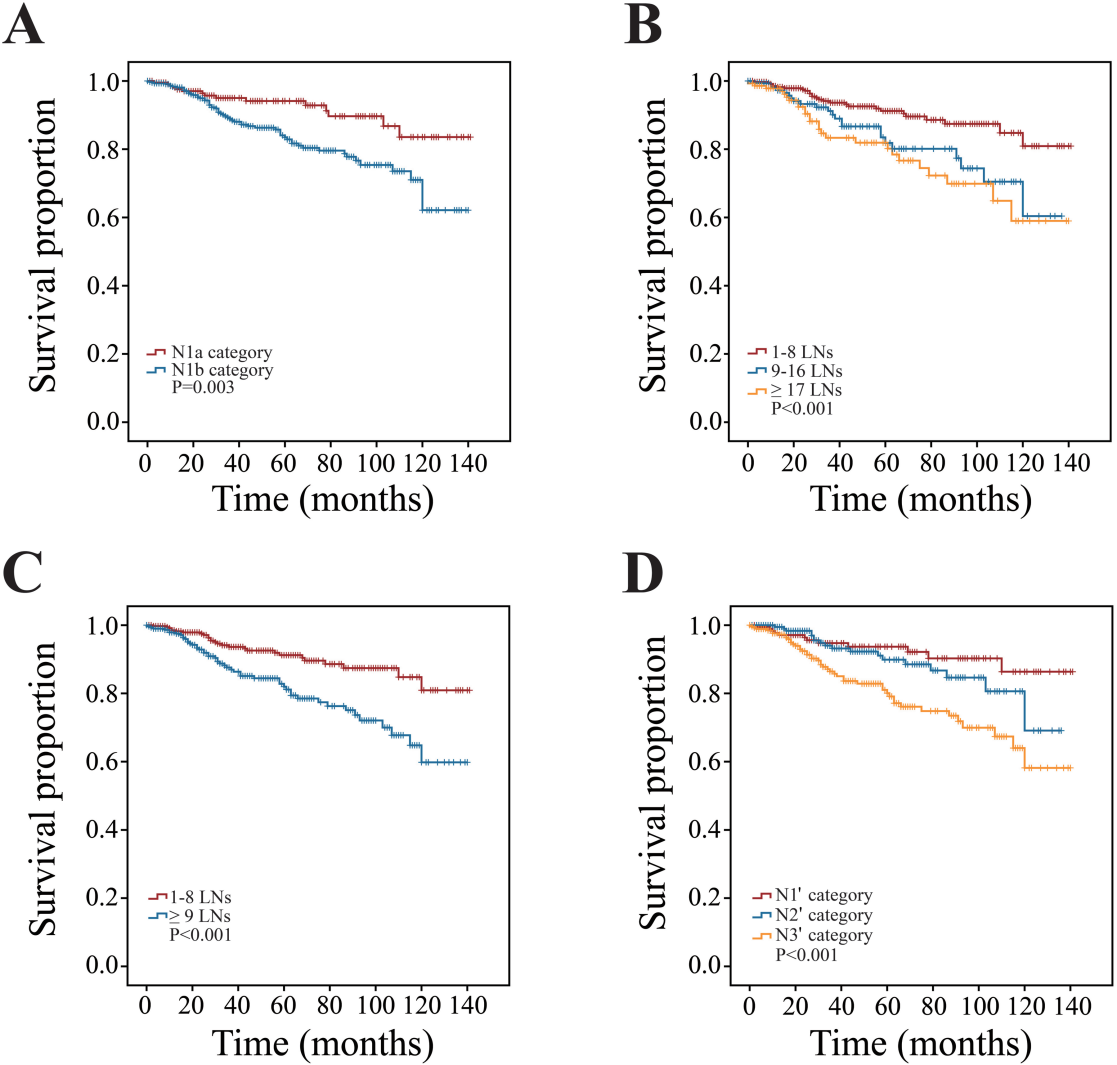
Kaplan-Meier survival curves stratified by the N category. (**A**) Survival curves of the N1a and N1b categories in the SEER database; (**B**) survival curves of 3 subgroups stratified by the LN count in the SEER database: 1-8 LNs, 9-16 LNs, and ≥17 LNs; (**C**) survival curves of 2 subgroups stratified by the LN count in the SEER database: 1-8 LNs and ≥9 LNs; and (**D**) survival curves stratified by the N1ʹ, N2ʹ, and N3ʹ categories in the SEER database.

Kaplan-Meier survival analysis showed that M0 patients with the N1b category had a worse prognosis than those with the N1a category (Fig. 2A, *P* = .003, HR = 2.271, 95%CI, 1.289-4.002). Further Chi-square test demonstrated that the N1b category harbored more numerous LNs than the N1a category (Table 2; *P < .*001). Accordingly, the discriminatory ability of the LN count was assessed. To achieve the greatest survival differences, X-tile software was applied to determine the optimal cutoff number of positive LNs (Fig. 2B; *P* = .001). M0 patients with LNM were divided into 3 groups: 1-8 LNs, 9-16 LNs, and ≥17 LNs. There was no significant survival difference between the group with 9-16 LNs and the group with ≥17 LNs (*P* = .339). Thus, in our analysis, the groups with 9-16 LNs and ≥17 LNs were combined into a single group (≥9 LNs). The group with ≥9 LNs experienced worse outcomes than the group with 1-8 LNs (Fig. 2C, *P* < .001, HR = 2.331, 95%CI, 1.457-3.729). These findings showed that both LN location and count could discriminate the prognosis of MTC patients well.

**Table 2.** Relationship between the LN location and count.

LN count	N1a category: metastasis to level VI or VII (paratracheal, paratracheal, or prelaryngeal/Delphian, or upper mediastinal) LNs. This can be unilateral or bilateral disease	N1b category: metastasis to unilateral, bilateral, or contralateral lateral neck LNs (levels I, II, III, IV, or V) or retropharyngeal LNs	N1ʹ category: N1a with 1-8 LNs	N2ʹ category: N1b with 1-8 LNs, N1a with ≥9 LNs	N3ʹ category: N1b with ≥9 LNs
1-4	149	93	149	93	—
5-8	34	85	34	85	—
9-12	17	77	—	17	77
13-16	6	58	—	6	58
17-20	1	46	—	1	46
21-24	3	27	—	3	27
≥25	3	62	—	3	62

Abbreviation: LN: lymph node.

Next, the intrinsic relationship among the T category, LN location, and LN count was studied. With T category upstaging (T1 to T4), the proportion of the N1b category showed a rising trend. The Chi-square test demonstrated that patients with advanced T stage (T3 + T4) had a higher proportion of N1b category than those with early T stage (T1 + T2) (*P* < .001), indicating that the T category was significantly associated with LN location. Furthermore, it was found that the proportion of N1b patients with ≥9 LNs in advanced T stages was nearly double that of early T stages (54.38% vs. 27.44%, *P* < .001), indicating that the T category was significantly associated with both LN location and count. Therefore, it is necessary to modify the current N category by combining the LN location and count as follows: N1a patients with 1-8 LNs were redefined as the N1ʹ category; N1a patients with ≥9 LNs and N1b patients with 1-8 LNs were redefined as the N2ʹ category; N1b patients with ≥9 LNs were redefined as the N3ʹ category. The N0’ category still referred to patients without LNM. Kaplan-Meier analysis showed significant survival differences among the 3 novel N categories (Fig. 2D, with N1ʹ category as reference, *P* < .001, HR of N3ʹ category = 3.062, 95%CI, 1.620-5.787), indicating the modified N category indeed had a stronger discriminatory capability.

### Modification of TNM Classification

**Figure 3.**
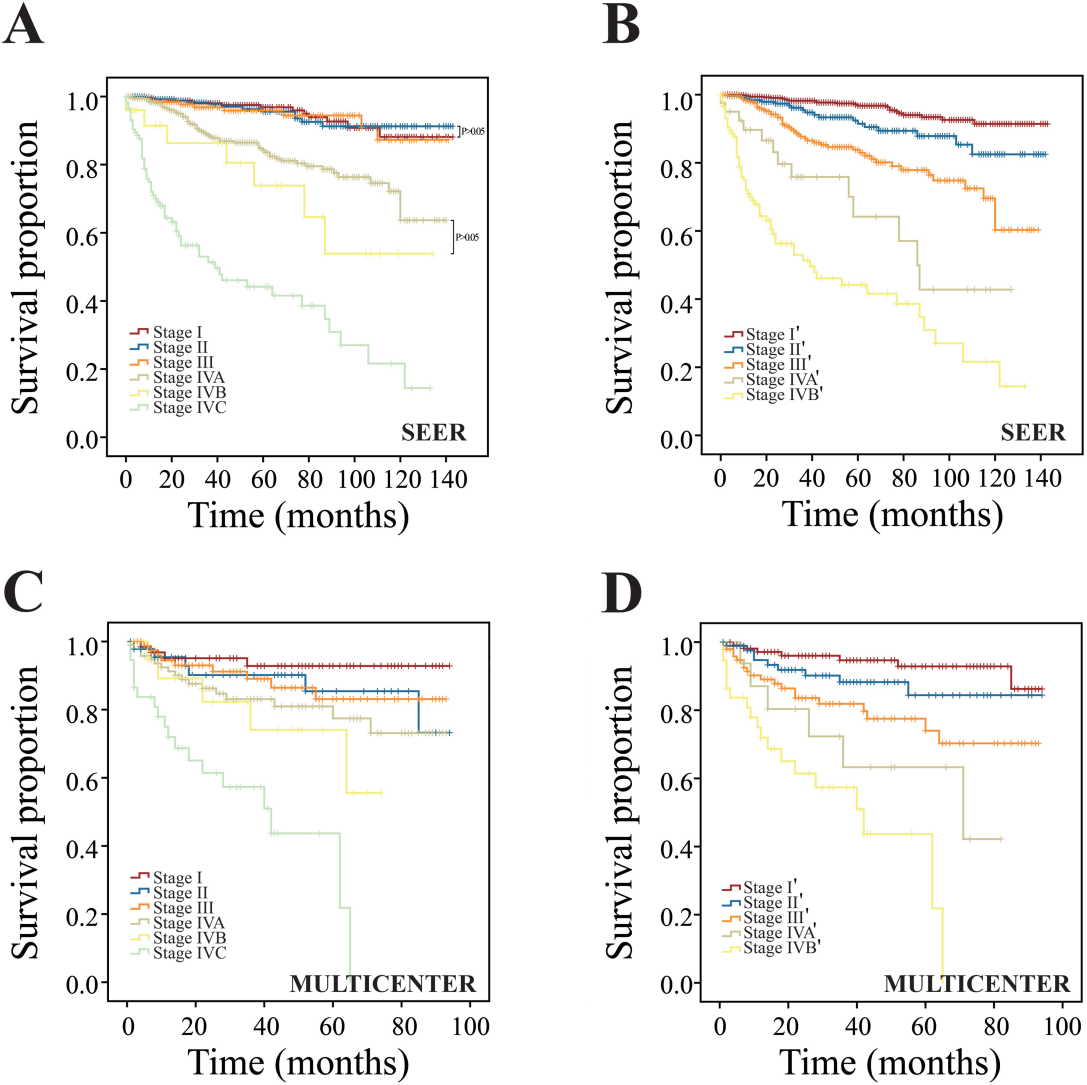
Kaplan-Meier survival curves according to 2 different staging systems. (**A**) Survival curves of the SEER cohort using the 8th AJCC staging system; (**B**) survival curves of the SEER cohort using the mAJCC staging system; (**C**) survival curves of the multicenter cohort using the 8th AJCC staging system; and (**D**) survival curves of the multicenter cohort using the mAJCC staging system.

According to the 8th edition of AJCC TNM staging criteria, no survival differences were observed among stages I, II, and III (Fig. 3A; *P* = .911). Patients with stage IVB had a shorter survival time than those with stage IVA, but statistical significance was not reached (Fig. 3A; *P* = .130). Therefore, we adopted the above novel T and N categories to modify the 8th TNM classification according to the principle of recursive partitioning analysis. The detailed modification was as follows: stage I (T1N0ʹ-2ʹM0, T2N0ʹM0), stage II (T1N3ʹM0, T2N1ʹ-2ʹM0, T3N0ʹ-1ʹM0), stage III (T2N3ʹM0, T3N2ʹ-3ʹM0, T4aʹN0ʹ-3ʹM0), stage IVA(T4bN0ʹ-3ʹM0), and stage IVB (T*any*N*any*M1) (Table 3). The results indicated that in the modified AJCC (mAJCC) staging system, survival differences between any 2 adjacent stages were significant (Fig. 3B; Table 4). The C-index for the mAJCC system was 0.811 (95%CI, 0.762-0.860) while that for the AJCC system was 0.792 (95%CI, 0.743-0.841), indicating that the mAJCC system showed better predictive power.

**Table 3.** T, N, and M category criteria in the 8th and modified AJCC staging system.

Eighth staging system		Modified staging system	
T category	T criteria	T category	T criteria
T0	No evidence of primary tumor	T0	Same as 8th
T1	Tumor ≤2 cm in greatest dimension limited to the thyroid	T1	Same as 8th
T2	Tumor >2 cm but ≤4 cm in greatest dimension limited to the thyroid	T2	Same as 8th
T3	Tumor >4 cm limited to the thyroid, or gross extrathyroidal extension invading only strap muscles	T3	Same as 8th
T4	Includes gross extrathyroidal extension	T4	Same as 8th
T4a	Gross extrathyroidal extension invading subcutaneous soft tissues, larynx, trachea, esophagus, or recurrent laryngeal nerve from a tumor of any size	T4aʹ	Gross extrathyroidal extension invading subcutaneous soft tissues, larynx, trachea, esophagus, recurrent laryngeal nerve, prevertebral fascia, carotid artery, or mediastinal vessels from a tumor ≤3.5 cm
T4b	Gross extrathyroidal extension invading prevertebral fascia or encasing the carotid artery or mediastinal vessels from a tumor of any size	T4bʹ	Gross extrathyroidal extension invading subcutaneous soft tissues, larynx, trachea, esophagus, recurrent laryngeal nerve, prevertebral fascia, carotid artery, or mediastinal vessels from a tumor >3.5 cm
**N category**	**N criteria**	**N category**	**N criteria**
N0	No evidence of locoregional lymph node metastasis	N0	Same as 8th
N1	Metastasis to regional nodes	N1ʹ	N1a category with 1-8 metastatic nodes
N1a	Metastasis to level VI or VII (paratracheal, paratracheal, or prelaryngeal/Delphian, or upper mediastinal) lymph nodes. This can be unilateral or bilateral disease	N2ʹ	(1) N1a category with ≥9 metastatic nodes (2) N1b category with 1-8 metastatic nodes
N1b	Metastasis to unilateral, bilateral, or contralateral lateral neck lymph nodes (levels I, II, III, IV, or V) or retropharyngeal lymph nodes	N3ʹ	N1b category with ≥9 metastatic nodes
**M category**	**M criteria**	**M category**	**M criteria**
M0	No distant metastasis	M0	Same as 8th
M1	Distant metastasis	M1	Same as 8th

Abbreviation: AJCC, American Joint Committee on Cancer.

**Table 4.** Comparison between the 8th AJCC and mAJCC staging system.

Stage	AJCC	HR (95%CI)	*P*-value	mAJCC	HR (95%CI)	*P*-value
I	T1N0M0			T1N0ʹ-2ʹM0 T2N0ʹM0		
II	T2-3N0M0	1.020 (0.471-2.209) with stage I as reference	.960 with stage I as reference	T1N3ʹM0 T2N1ʹ-2ʹM0 T3N0ʹ-1ʹM0	2.280 (1.249-4.162) with stage I as reference	.007 with stage I as reference
III	T1-3N1aM0	1.176 (0.495-2.793) with stage II as reference	.714 with stage II as reference	T2N3ʹM0 T3N2ʹ-3ʹM0 T4aʹN0ʹ-3ʹM0	2.248 (1.317-3.839) with stage II as reference	.003 with stage II as reference
IVA	T1-3N1bM0 T4aN*any*M0	3.364 (1.667-6.789) with stage III as reference	.001 with stage III as reference	T4bʹN0ʹ-3ʹM0	2.417 (1.305-4.477) with stage III as reference	.005 with stage III as reference
IVB	T4bN*any*M0	1.833 (0.837-4.013) with stage IVA as reference	.130 with stage IVA as reference	T*any*N*any*M1	2.107 (1.150-3.860) with stage IVA as reference	.016 with stage IVA as reference
IVC	T*any*N*any*M1	2.789 (1.264-6.158) with stage IVB as reference	.011 with stage IVB as reference			

Abbreviations: AJCC, American Joint Committee on Cancer; HR: hazard ratio.

### Multicenter Validation

There was a total of 349 MTC patients in the multicenter dataset between 2010 and 2018 (Table 1). All of them were Chinese. According to the 8th AJCC TNM staging system, 65 cases had stage I, 46 had stage II, 87 had stage III, 95 had stage IVA, 19 had stage IVB, and 37 had stage IVC disease. Tumor size ranged from 3 to 85 mm with a median size of 20 mm. The number of LNM ranged from 0 to 42 with the median number of 2. Survival analysis revealed that the mAJCC system was more effective at distinguishing the prognosis (Fig. 3C, 3D). The C-index for the mAJCC system was 0.725 (95%CI, 0.629-0.821) while that for the AJCC system was 0.686 (95%CI, 0.588-0.784).

## Discussion

In the 8th edition of the AJCC staging system, the T4 category was divided into T4a and T4b based on the degree of tumor invasion. But we found there were no significant survival differences between T4a and T4b categories. Although a retrospective study from Portugal et al showed that the T4b category was associated with a worse prognosis, the difference did not reach statistical significance (*P* = .06).^18^ In our study, the division of the T4 category based on the tumor size could better stratify the death risk of MTC patients (*P* = .003). This implicated that tumor size played a significant role not only in the T1-3 category but also in the T4 category. To the best of our knowledge, this is the first study to improve the T4 category of MTC patients, as well as to provide the foundation for significantly distinguishing individuals with stage IVA and those with stage IVB in the current staging system.

Mohamed et al^9^ improved the current TNM staging system based on the principle of maximum overall survival differences. Mathiesen et al^19^ used Danish National Cancer Database to further validate the proposed staging system by Mohamed et al and found that it indeed provided better survival discrimination than the current staging system. However, there were several main shortcomings in the proposal by Mohamed et al. First, the proposed staging system could differentiate the prognosis of patients with stages I, II, and III in the training cohort (NCDB data) but not in the validation cohort (SEER data), indicating that the proposed staging system might not enjoy universal applicability. Comparatively, our mAJCC staging system showed a fair performance in both the training and validation cohort, especially using the SEER data. Second, the T4 category was subdivided into T4a and T4b in the current AJCC staging system, while the T4 category was considered as a whole in the proposed staging system, which partially masked the tumor heterogeneity and reduced the discriminatory power to some extent. We improved the T4 category and yielded encouraging results. Third, patients with stage IV were not further divided into subgroups in the proposed staging system by Mohamed et al, which would make refined management of MTC patients more challenging. The same problem existed in the study by Jes et al.^19^ In our analyses, the subdivision of patients with stage IV based on the novel T4 category addressed the issue of the huge “survival gap” between stages III and IV and made the survival curves more evenly distributed. As shown in Table 4, there was an approximately 2-fold increase in the risk of death between each 2 adjacent staging groups.

LNM was frequent in MTC and more than half of the cases developed LNM at the time of diagnosis.^20,21^ The LN location was an independent risk factor for MTC and was used as a classification criterion in the current AJCC staging system.^8,22^ In recent years, an increasing number of studies have shown that having more LNs was independently associated with worse outcomes in MTC patients.^14,23,24^ These findings highlighted the crucial role of the LN count. A large retrospective study from Korea suggested that the mean positive LN number of the N1a category was much lower than that of the N1b category (0.3 vs. 12.8), which showed a significant intrinsic relationship between the LN location and count.^25^ Their results were consistent with our findings. The combination of the LN location and count allowed a more detailed risk-stratification, which was also the key to discriminate patients with stages I, II, and III. For example, T1-3N1aM0 was collectively classified as stage III in the 8th AJCC staging system. However, in the mAJCC staging system, T1N0ʹ-2ʹM0 was classified as stage I, T2N1ʹ-2ʹM0 and T3N1ʹM0 as stage II, and T3N2ʹM0 as stage III. It was discovered that the modified staging system performed well.

There were some limitations in the present study. First, the most obvious limitation was the retrospective nature. Future prospective studies with large sample sizes were needed to validate the accuracy and efficiency of the mAJCC staging system. Second, the SEER database lacks some important information such as calcitonin, carcinoembryonic antigen (CEA), mutation status of *RET*, and disease recurrence and progression, which eliminated the possibility of evaluating their effects on the staging system. Several studies have indicated that increased CEA and calcitonin levels were associated with larger tumors and more metastatic LNs, suggesting a larger extent of surgery.^26,27^ These biochemical parameters also had the potential for predicting MTC recurrence.^28^*RET* gene mutation is an important molecular event during the development and progression of MTC and has been implicated in LNM of MTC and disease persistence following surgery.^29^ In future updates of the AJCC staging system for MTC, the inclusion of key biochemical and genetic considerations will be an important direction. Third, the data quality of the multicenter database may be inferior to that of the SEER database, resulting in a lower value of C-index. Fourth, a minority of patients had small tumors, which might be diagnosed incidentally during surgery. The incidental MTC might result in an inadequate extent of surgical resection and thereby unfavorable clinical outcomes.^30^ There is also an increased risk of missed diagnosis of pheochromocytoma and hyperparathyroidism.^31^

## Conclusions

In summary, we improved the 8th AJCC staging system based on the intrinsic relationship among the T category, LN location, and LN count. The mAJCC staging system showed a better performance than the 8th AJCC staging system, which would be beneficial to clinical decision-making and appropriate surveillance.

## Data Availability

Datasets analyzed during the present study can be made available from the corresponding author on reasonable request.

## References

[1] Noone AM , Cronin KA, Altekruse SF, et al. Cancer incidence and survival trends by subtype using data from the surveillance epidemiology and end results program, 1992-2013. Cancer Epidemiol Biomarkers Prev. 2017;26(4):632-641. 10.1158/1055-9965.EPI-16-0520. 27956436 PMC5380602

[2] Viola D , Elisei R. Management of medullary thyroid cancer. Endocrinol Metab Clin North Am. 2019;48(1):285-301. 10.1016/j.ecl.2018.11.006. 30717909

[3] Bhoj VG , Li L, Parvathaneni K, et al. Adoptive T cell immunotherapy for medullary thyroid carcinoma targeting GDNF family receptor alpha 4. Mol Ther Oncolytics. 2021;20:387-398. 10.1016/j.omto.2021.01.012. 33614919 PMC7879023

[4] Shi X , Li CW, Tan LC, et al. Immune co-inhibitory receptors pd-1, ctla-4, tim-3, lag-3, and tigit in medullary thyroid cancers: a large cohort study. J Clin Endocrinol Metab. 2021;106(1):120-132. 10.1210/clinem/dgaa701. 33000173

[5] Almeida MQ , Hoff AO. Recent advances in the molecular pathogenesis and targeted therapies of medullary thyroid carcinoma. Curr Opin Oncol. 2012;24(3):229-234. 10.1097/CCO.0b013e328351c71a. 22343387

[6] Lacouture ME , Ciccolini K, Kloos RT, Agulnik M. Overview and management of dermatologic events associated with targeted therapies for medullary thyroid cancer. Thyroid. 2014;24(9):1329-1340. 10.1089/thy.2013.0700. 24902006 PMC4148058

[7] Aschebrook-Kilfoy B , Ward MH, Sabra MM, Devesa SS. Thyroid cancer incidence patterns in the united states by histologic type, 1992–2006. Thyroid. 2011;21(2):125-134. 10.1089/thy.2010.0021. 21186939 PMC3025182

[8] Amin MB , Edge S, Greene F, Byrd DR, Brookland RK, Washington MK. Ajcc Cancer Staging Manual. 8th ed. New York, NY: Springer; 2017

[9] Adam MA , Thomas S, Roman SA, Hyslop T, Sosa JA. Rethinking the current American joint committee on cancer TNM staging system for medullary thyroid cancer. JAMA Surg. 2017;152(9):869-876. 10.1001/jamasurg.2017.1665. 28636692 PMC5710458

[10] Chen L , Qian K, Guo K, et al. A novel N staging system for predicting survival in patients with medullary thyroid cancer. Ann Surg Oncol. 2019;26(13):4430-4438. 10.1245/s10434-019-07871-1. 31552613

[11] Le Voyer TE , Sigurdson ER, Hanlon AL, et al. Colon cancer survival is associated with increasing number of lymph nodes analyzed: a secondary survey of intergroup trial int-0089. J Clin Oncol. 2003;21(15):2912-2919. 10.1200/JCO.2003.05.062. 12885809

[12] Ding D , Stokes W, Eguchi M, et al. Association between lymph node ratio and recurrence and survival outcomes in patients with oral cavity cancer. JAMA Otolaryngol Head Neck Surg. 2019;145(1):53-61. 10.1001/jamaoto.2018.2974. 30452499 PMC6439806

[13] Wang M , Hu D, Zeng W, et al. New proposed tumor-node-metastasis staging system for medullary thyroid carcinoma based on the surveillance, epidemiology, and end results database. Am J Transl Res. 2020;12(6):2703-2710. 32655802 PMC7344094

[14] Machens A , Dralle H. Prognostic impact of n staging in 715 medullary thyroid cancer patients: proposal for a revised staging system. Ann Surg. 2013;257(2):323-329. 10.1097/SLA.0b013e318268301d. 22968075

[15] Yang JH , Lindsey SC, Camacho CP, et al. Integration of a postoperative calcitonin measurement into an anatomical staging system improves initial risk stratification in medullary thyroid cancer. Clin Endocrinol (Oxf). 2015;83(6):938-942. 10.1111/cen.12657. 25376110

[16] Wang Z , Tang C, Wang Y, Yin Z, Rixiati Y. Inclusion of the number of metastatic lymph nodes in the staging system for medullary thyroid cancer: validating a modified American joint committee on cancer tumor-node-metastasis staging system. Thyroid. 2022;32(5):536-543. 10.1089/thy.2021.0571. 35350868

[17] von Elm E , Altman DG, Egger M, et al; STROBE Initiative. Strengthening the reporting of observational studies in epidemiology (strobe) statement: guidelines for reporting observational studies. BMJ. 2007;335(7624):806-808. 10.1136/bmj.39335.541782.AD. 17947786 PMC2034723

[18] Peixoto Callejo I , Americo Brito J, Zagalo CM, Rosa Santos J. Medullary thyroid carcinoma: multivariate analysis of prognostic factors influencing survival. Clin Transl Oncol. 2006;8(6):435-443. 10.1007/s12094-006-0198-2. 16790397

[19] Mathiesen JS , Kroustrup JP, Vestergaard P, et al. Replication of newly proposed TNM staging system for medullary thyroid carcinoma: a nationwide study. Endocr Connect. 2019;8(1):1-7. 10.1530/EC-18-0494. 30550378 PMC6330714

[20] Jin LX , Moley JF. Surgery for lymph node metastases of medullary thyroid carcinoma: a review. Cancer. 2016;122(3):358-366. 10.1002/cncr.29761. 26539937

[21] Moley JF. Medullary thyroid carcinoma: management of lymph node metastases. J Natl Compr Canc Netw. 2010;8(5):549-556. 10.6004/jnccn.2010.0042. 20495084

[22] Rozenblat T , Hirsch D, Robenshtok E, et al. The prognostic value of lymph node ratio in medullary thyroid carcinoma: a multi-center study. Eur J Surg Oncol. 2020;46(11):2023-2028. 10.1016/j.ejso.2020.04.016. 32389525

[23] Kebebew E , Ituarte PH, Siperstein AE, Duh QY, Clark OH. Medullary thyroid carcinoma: Clinical characteristics, treatment, prognostic factors, and a comparison of staging systems. Cancer. 2000;88(5):1139-1148. 10.1002/(sici)1097-0142(20000301)88:5<1139::aid-cncr26>3.0.co;2-z. 10699905

[24] Esfandiari NH , Hughes DT, Yin H, Banerjee M, Haymart MR. The effect of extent of surgery and number of lymph node metastases on overall survival in patients with medullary thyroid cancer. J Clin Endocrinol Metab. 2014;99(2):448-454. 10.1210/jc.2013-2942. 24276457 PMC3913800

[25] Park H , Park J, Choi MS, et al. Preoperative serum calcitonin and its correlation with extent of lymph node metastasis in medullary thyroid carcinoma. Cancers (Basel). 2020;12(10):2894-2894. 33050233 10.3390/cancers12102894 PMC7601718

[26] Ye L , Zhou X, Lu J, et al. Combining serum calcitonin, carcinoembryonic antigen, and neuron-specific enolase to predict lateral lymph node metastasis in medullary thyroid carcinoma. J Clin Lab Anal. 2020;34(7):e23278. 10.1002/jcla.23278. 32141647 PMC7370728

[27] Machens A , Dralle H. Biomarker-based risk stratification for previously untreated medullary thyroid cancer. J Clin Endocrinol Metab. 2010;95(6):2655-2663. 10.1210/jc.2009-2368. 20339026

[28] Wolinski K , Kaznowski J, Klimowicz A, et al. Diagnostic value of selected biochemical markers in the detection of recurrence of medullary thyroid cancer—comparison of calcitonin, procalcitonin, chromogranin a, and carcinoembryonic antigen. Endokrynol Pol. 2017;68(4):434-437. 10.5603/EP.a2017.0038. 28585679

[29] Accardo G , Conzo G, Esposito D, et al. Genetics of medullary thyroid cancer: an overview. Int J Surg. 2017;41(Suppl 1):S2-S6. 10.1016/j.ijsu.2017.02.064. 28506408

[30] Haddad RI , Bischoff L, Ball D, et al. Thyroid carcinoma, version 2.2022, NCCN clinical practice guidelines in oncology. J Natl Compr Canc Netw. 2022;20(8):925-951. 10.6004/jnccn.2022.0040. 35948029

[31] Ahmed SR , Ball DW. Clinical review: incidentally discovered medullary thyroid cancer: diagnostic strategies and treatment. J Clin Endocrinol Metab. 2011;96(5):1237-1245. 10.1210/jc.2010-2359. 21346073 PMC3085196

